# Supplementary material for: Magnetic Resonance Imaging as a Prognostic Disability Marker in Clinically Isolated Syndrome and Multiple Sclerosis: A Systematic Review and Meta-Analysis
Source: Diagnostics (Basel). 2022 Jan 21;12(2):270. doi: 10.3390/diagnostics12020270 (PMC8871297; doi:10.3390/diagnostics12020270)
Supplement: Supplementary file 1 [file diagnostics-12-00270-s001.zip › diagnostics-1541057-supplementary.pdf]

Supplementary Figure S1. Search strategy and key works (assessed by librarians)

Web of science
("multiple sclerosis" OR "MS") AND ("Magnetic resonance imaging" OR "MRI") AND (white matter lesion OR lesions) AND (long*) AND (Disability) AND (T2 or FLAIR)

PubMed
("multiple sclerosis" OR "MS") AND ("Magnetic resonance imaging" OR "MRI") AND (white matter lesion OR lesions) AND (long*) AND (Disability) AND (T2 or FLAIR)

Embase:
("multiple sclerosis"[MeSH Terms] OR "multiple sclerosis"[All Fields] OR "MS"[All Fields]) AND ("magnetic resonance imaging"[MeSH Terms] OR "magnetic"[All Fields] OR "imaging"[All Fields]) AND ("lesion" [All Fields] OR "lesions"[All Fields])
AND (white matter [All Fields]) AND ("disability"[All Fields]) AND (long* [All Fields]) AND ("T2" [All Fields] OR "FLAIR" [All Fields])

Below is a sample search I ran in Ovid MEDLINE. Notice that both MeSH terms *and* keywords (the lines ending in **.mp.**) have been used. The MeSH terms have been 'exploded' (**exp** Magnetic Resonance Imaging/) and included all their sub-headings. The number of results I got was **102**

1	exp Magnetic Resonance Imaging/	470430
2	(MRI or "magnetic resonance imaging").mp. [mp=title, abstract, original title, name of substance word, subject heading word, floating sub-heading word, keyword heading word, organism supplementary concept word, protocol supplementary concept word, rare disease supplementary concept word, unique identifier, synonyms]	603156
3	(WML or "white matter lesion*").mp. [mp=title, abstract, original title, name of substance word, subject heading word, floating sub-heading word, keyword heading word, organism supplementary concept word, protocol supplementary concept word, rare disease supplementary concept word, unique identifier, synonyms]	5290
4	(count* or number*).mp. [mp=title, abstract, original title, name of substance word, subject heading word, floating sub-heading word, keyword heading word, organism supplementary concept word, protocol supplementary concept word, rare disease supplementary concept word, unique identifier, synonyms]	3384137
5	volume*.mp. [mp=title, abstract, original title, name of substance word, subject heading word, floating sub-heading word, keyword heading word, organism supplementary concept word, protocol supplementary concept word, rare disease supplementary concept word, unique identifier, synonyms]	771624
6	exp Multiple Sclerosis/	60809
7	(MS or "multiple sclerosis").mp. [mp=title, abstract, original title, name of substance word, subject heading word, floating sub-heading word, keyword heading word, organism supplementary concept word, protocol supplementary concept word, rare disease supplementary concept word, unique identifier, synonyms]	400696
8	exp Disability Evaluation/	53879
9	(EDSS or "Expanded Disability Status Scale").mp. [mp=title, abstract, original title, name of substance word, subject heading word, floating sub-heading word, keyword heading word, organism supplementary concept word, protocol supplementary concept word, rare disease supplementary concept word, unique identifier, synonyms]	6415
10	(disabilit* or disabled).mp. [mp=title, abstract, original title, name of substance word, subject heading word, floating sub-heading word, keyword heading word, organism supplementary concept word, protocol supplementary concept word, rare disease supplementary concept word, unique identifier, synonyms]	334935
11	1 or 2	623201
12	4 or 5	4032476
13	6 or 7	400696
14	8 or 9 or 10	339822
15	3 and 11 and 12 and 13 and 14	102

Supplementary Table S1. MRI data, raters, and lesion segmentation.

Study	T2 Lesion Sequence	T2/PD Slice Thickness	MRI -Tesla	Blinded to Clinical Details	No. of raters	Lesion Segmentation	Software
Tintore 2020	T2, PD, FLAIR	3-5mm	1.5T or 3T	⃠	⃠	⃠	⃠
Chung 2020	T2, PD	5-10mm	1.5T or 3T	⃠	3	⃠	⃠
Brownlee,2019	T2, PD	3mm	1.5T	Yes	1	Semi-automated	JIM6, Xinapse systems, Aldwincle, UK
Tintore 2015*	T2, PD, Flair	3-5 mm	1.5T or 3T	⃠	⃠	⃠	⃠
Jacobsen 2014*	T2, PD	5mm	1.5T	⃠	⃠	Semi-automated	⃠
Kearney 2014	T2	1-3mm	1.5T or 3T	⃠	2	Semi-automated	JIM6, Xinapse systems, Aldwincle, UK
Giorgio 2014	T2, PD	3mm	1.5T	Yes	1	Semi-automated	Jim 5.0, Xinapse System, Leicester, UK
Popescu 2013	T2	3-5mm	1T or 1.5T	Yes	1	Semi-automated	Jim 5.0, Xinapse System, Leicester, UK
Rovaris 2011	T2	3-5mm	1T or 1.5T	Yes	1	Semi-automated	Jim, Xinapse System, Leicester, UK
Renard 2010	T2, PD, Flair	3mm	1.5T	Yes	2	Semi-automated	⃠
Fisniku 2008*	T2	5-10mm	0.5T	Yes	1	Semi-automated	DispImage
Chard 2003	T2	5-10mm	1.5T	Yes	2	Semi-automated	DispImage
Brex. 2002	T2	5-10mm	0.5T	⃠	⃠	Semi-automated	DispImage
Sailer 1999	T2	5-10mm	0.5	⃠	1	Semi-automated	DispImage
O'Riordan 1998	⃠	5-10mm	0.5	Yes	2	Semi-automated	DispImage
**Reported (%)**	**14 (93%)**	**15 (100%)**	**15 (100%)**	**8 (53%)**	**11 (73%)**	**12 (80%)**	**10 (66%)**

⃠: not reported, T2: T2 weighted images, PD: proton density, *studies included in the Mata-analysis, No: Number.

Supplementary Figure S2. Key elements for forest plot interpretation.

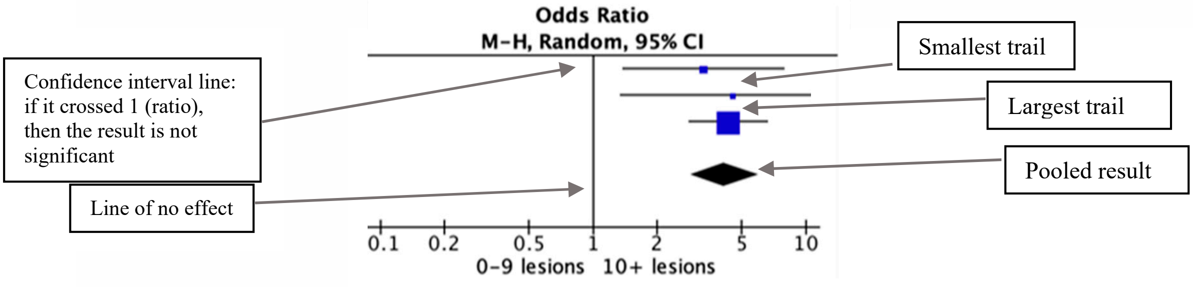

Legend:

This square represents the individual studies effect. The size varies to reflect the weight a specific study has in the overall analysis (larger “symbol” has more weight).

The black line represents the Cis of the study; the smaller “symbol” which has less weight generally has larger Cis than the larger “symbol”.

The diamond represents the overall or summary effect. The outer edges of the diamond represent the CIs.
